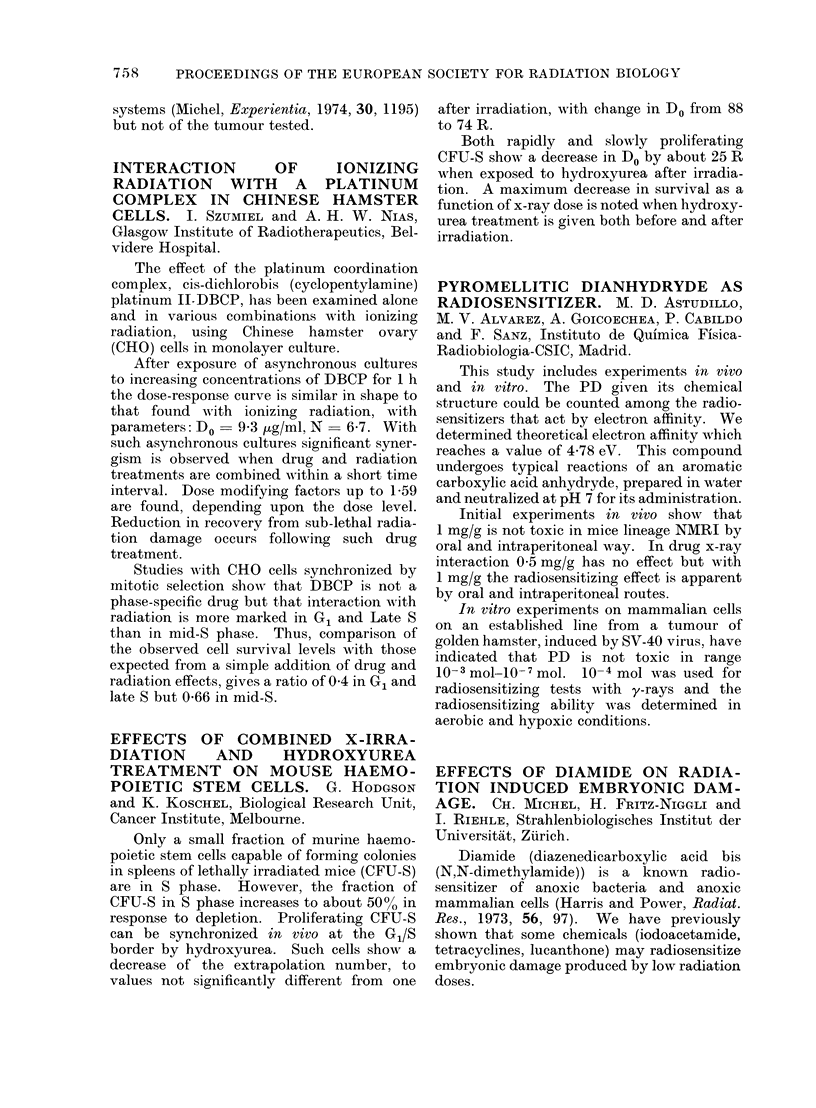# Proceedings: Effects of combined x-irradiation and hydroxyurea treatment on mouse haemopoietic stem cells.

**DOI:** 10.1038/bjc.1975.312

**Published:** 1975-12

**Authors:** G. Hodgson, K. Koschel


**EFFECTS OF COMBINED X-IRRADIATION AND HYDROXYUREA TREATMENT ON MOUSE HAEMOPOIETIC STEM CELLS.** G. HODGSON and K. KOSCHEL, Biological Research Unit, Cancer Institute, Melbourne.

Only a small fraction of murine haemopoietic stem cells capable of forming colonies in spleens of lethally irradiated mice (CFU-S) are in S phase. However, the fraction of CFU-S in S phase increases to about 50% in response to depletion. Proliferating CFU-S can be synchronized *in vivo* at the $G_1/S$ border by hydroxyurea. Such cells show a decrease of the extrapolation number, to values not significantly different from one after irradiation, with change in $D_0$ from 88 to 74 R.

Both rapidly and slowly proliferating CFU-S show a decrease in $D_0$ by about 25 R when exposed to hydroxyurea after irradiation. A maximum decrease in survival as a function of x-ray dose is noted when hydroxyurea treatment is given both before and after irradiation.